# Pulfrich's stereo curtain

**DOI:** 10.1177/20416695251338727

**Published:** 2025-05-27

**Authors:** Stuart M Anstis, Joshua A Solomon, Christopher W Tyler

**Affiliations:** 1 Department of Psychology, University of California, San Diego, La Jolla, CA, USA; 2 4895 City University of London, London, UK; 3 70042 Smith-Kettlewell Eye Research Institute, San Francisco, CA, USA

**Keywords:** motion, stereo vision, binocular vision, surfaces

## Abstract

Travelling longitudinal waves provide a novel visual motion stimulus that supports the perception of stereoscopic travelling waves from a phased array of sinusoidal oscillations.

## How to cite this article

Anstis, S. M., Solomon, J. A., & Tyler, C. W. (2025). Pulfrich's stereo curtain. *i-Perception, 16*(0), 1–5. https://doi.org/10.1177/20416695251338727

In longitudinal waves such as sound waves, each particle or molecule does not drift along with the wave, but instead oscillates back and forth about a fixed equilibrium position. The wave can thus be considered an emergent property, drifting to the right if each molecule responds with a slight delay or phase lag compared to its left-hand neighbor. Inspired by the on-line resource created by Zeleny et al. (2011), we created a longitudinal wave from a set of vertical lines oscillating sinusoidally in place—a form of first-order local motion—by giving them a progressive temporal phase lag across the array (see Figure 1). The wave can be perceived as regions of compression and rarefaction that drift steadily to the right in an emergent form of global motion. This form of wave is a novel higher-order percept.

**Figure 1.** A travelling wave of line oscillations from the progressive temporal phase lag across the array. Double-click to activate the wave motion. Cross the eyes to see three red Xs in order to obtain the stereoscopic view of the wave. [In this and the following figures, view the corresponding Movie File.]

Specifically, ten vertical lines define each wavelength, so each line has a phase lag of 36° (=360°/10) with respect to its left-hand neighbor. A total phase lag of 360° across the 10 lines defines the wavelength of the longitudinal travelling wave. Attention is switchable at will between the local oscillations and the travelling-wave percepts, but they cannot both be seen simultaneously.

Figure 2 is a control stimulus showing the same array as Figure 1 with the same local oscillations of each line, but all in the same phase to give the whole array a fixed oscillation rather than a travelling wave.

**Figure 2.** An oscillatory wave motion with the same local oscillations. This is as in Figure 1, but all at the same temporal phase to form rigid motion.

To obtain a stereoscopic view of this wave in depth, one could look at these displays with both eyes open but with a neutral density filter over your left eye. Each line would act as a little Pulfrich's Pendulum, and instead of lying in the plane of the paper would appear to follow an elliptical orbit, coming forward out of the paper as it moves left and going back behind the paper as it moves to the right (Pulfrich, 1922). This would make the whole surface of the page look corrugated in depth (like a piece of corrugated roofing with its bars running vertically: see Bradshaw & Rogers, 1999). The denser the filter, the deeper the apparent corrugations (Anstis & Rogers, 1972). Now throw away the ND filter because it is not necessary. Instead, simply look at the two X's on the left, which are horizontally separated by 0.2 of a wavelength, a phase shift of 72°, and cross your eyes until you see three X's (not two or four). Each line will now appear to move around an elliptical orbit in depth, and as a result, the whole surface appears corrugated, with the rarefied regions apparently standing out in front and the compressed regions lying behind. Crossing the eyes is like looking at an autostereogram (Ninio 2007; Tyler, 1983; Tyler & Clarke, 1990), also known commercially as Magic Eye 3D illusions. When the travelling wave is viewed with convergence, it will appear to float in the plane of fixation where the lines of sight from the two eyes cross in front of the screen and will consequently look smaller than in direct view (Emmert's law). Conversely, if the lines of sight are diverged to see the three crosses behind the plane of the screen, the image will float behind the screen and appear to be larger than in direct view.

In a second version of the travelling wave (Figure 3), the oscillating lines are broken up into short vertical segments to provide more line-end cues for the cortical processing (see Figures 3 and 4), and hence may support a stronger depth percept.

**Figure 3.** An oscillatory wave motion with the same local oscillations. This is as in Figure 1, but with the lines broken up.

**Figure 4.** The control fixed-phase version of Figure 3, giving rigid motion.

A further modification is shown in Figures 5 and 6, where the local motion is drift-balanced because the background consists of equally-spaced black and white bars, and the moving targets are black-white doublets of twice the width of the bars, thus in both cases forming 100% contrast against a net 50% gray background. As in Figures 1 and 3, the drift-balanced wave in Figure 4 can be perceived as regions of compression and rarefaction that drift steadily to the right, and the rigid motion in Figure 6 shows the corresponding uniform-phase version, as in Figure 2.

**Figure 5.** Drift-balanced version of the travelling wave of line segment oscillations (compare with Figure 3).

**Figure 6.** The control fixed-phase version of Figure 5.

Although longitudinal wave motion is a familiar concept in the physics of compression waves, it does not seem to have been studied as a visual perceptual phenomenon. The visual presentation of the compression waves in drift-balanced form (Figure 5) shows that this wave motion is perceptible, even when balanced for first-order luminance motion.

In conclusion, the classic Pulfrich effect—viewing a moving target with a neutral density filter over one eye—tells us something new about the visual system—visual latencies vary as a function of luminance (Rogers & Anstis, 1972). Our demonstrations combine visual principles that are already well-known—depth from disparity, motion, and autostereograms—to produce a novel form of travelling wave that is derivative of, but perceptually distinct from, the Pulfrich-like oscillations. And whereas Pulfrich had a single point into depth, we give the depth structure of a large array of points an emergent higher-order travelling-wave motion signal. All movies can be viewed at: https://www.dropbox.com/scl/fo/56mruz79qlpqnros39gei/AHbnar20k1RGSBSgSyQvYI8?rlkey=nf8sppm5k7ud08gnsvq1h43d7&st=ibfh8zum&dl=0.
